# Use of a Cancer Registry to Evaluate Patient-Reported Outcomes of Immune Checkpoint Inhibitors

**DOI:** 10.3390/cancers13010103

**Published:** 2020-12-31

**Authors:** Heather S. L. Jim, Sarah L. Eisel, Aasha I. Hoogland, Sandra Shaw, Jennifer C. King, Adam P. Dicker

**Affiliations:** 1 Moffitt Cancer Center, Department of Health Outcomes and Behavior, Tampa, FL 33612, USA; Sarah.Eisel@moffitt.org (S.L.E.); Aasha.Hoogland@moffitt.org (A.I.H.); 2 GO_2_ Foundation for Lung Cancer, Washington, DC 20006, USA; sshaw@mjhlifesciences.com (S.S.); jking@go2foundation.org (J.C.K.); 3 Jefferson Center for Digital Health, Department of Radiation Oncology, Thomas Jefferson University, Philadelphia, PA 19107, USA; Adam.Dicker@jefferson.edu

**Keywords:** antineoplastic agents, immunological, programmed cell death receptor 1, lung neoplasms, quality of life, patient-reported outcome measures

## Abstract

**Simple Summary:**

Immune checkpoint inhibitors (ICIs) are increasingly used for advanced lung cancer, but few studies have reported on patient-reported outcomes (PROs) outside the context of a clinical trial. The goal of the current study was to assess PROs in patients participating in the GO_2_ Foundation’s Lung Cancer Registry who reported receiving atezolizumab, durvalumab, nivolumab, or pembrolizumab. Internationally, 226 patients (mean age 61, 75% female) participated. Patients reported worse quality of life than U.S. population and cancer normative samples. The most common moderate to severe adverse events during ICI treatment were fatigue (41%), aching joints (27%), and aching muscles (20%). Due to toxicity, 25% reported a treatment delay, 11% an emergency room visit, and 9% a hospitalization. This study is among the first to our knowledge to report on PROs of ICIs outside the context of a clinical trial. Results suggest higher rates of adverse events than previously reported in clinical trials.

**Abstract:**

Immune checkpoint inhibitors (ICIs) are increasingly used for advanced lung cancer, but few studies have reported on patient-reported outcomes (PROs) outside the context of a clinical trial. The goal of the current study was to assess PROs in participants of a lung cancer registry who had been treated with an ICI. Patients participating in the GO_2_ Foundation’s Lung Cancer Registry who reported receiving atezolizumab, durvalumab, nivolumab, or pembrolizumab were invited to participate in a survey about their experiences during treatment. Quality of life was evaluated using the Functional Assessment of Cancer Therapy–General (FACT-G). Common symptomatic adverse events were evaluated using an item bank generated for ICIs. Internationally, 226 patients (mean age 61, 75% female) participated. Patients reported worse quality of life at the time of assessment than U.S. population and cancer normative samples. The most common moderate to severe adverse events during ICI treatment were fatigue (41%), aching joints (27%), and aching muscles (20%). Due to toxicity, 25% reported a treatment delay, 11% an emergency room visit, and 9% a hospitalization. This study is among the first to our knowledge to report on PROs of ICIs outside the context of a clinical trial. Results suggest higher rates of adverse events than previously reported in clinical trials.

## 1. Introduction

Immune checkpoint inhibitors (ICIs) have generated widespread excitement for their ability to significantly prolong survival in cancers with poor prognoses. Non-small-cell lung cancer (NSCLC) has emerged as a target of immune-based therapies, with some patients with advanced NSCLC experiencing durable remissions and prolonged survival [1,2].

The success of ICIs in slowing disease progression comes with the cost of toxicity, however. All checkpoint inhibitors can potentially induce immune-related toxicities in any organ system. Immune-related toxicities occur in up to 70% of patients treated with PD-1/PD-L1 antibodies [3,4]. Up to 16% of patients experience grade 3 or higher toxicities [3,4]. The single-agent anti-PD-1 antibodies, such as nivolumab and pembrolizumab, have fewer side effects than combination ipilimumab/nivolumab (grade 3 or 4 adverse events in 10–15% versus grade 3 or 4 adverse events in 55%). Common toxicities of PD-1/PD-L1 antibodies include fatigue, diarrhea, rash, pruritus, decreased appetite, hepatic toxicities, and endocrinopathies [3,4].

There is increasing awareness of the importance of collecting patient-reported outcome (PRO) data in oncology [5,6,7]. PROs can be defined as “reports of the status of a patient’s health condition that come directly from the patient, without interpretation of the patient’s response by a clinician or anyone else” [5]. PROs can be categorized as disease symptomatology, treatment side effects, and quality of life (i.e., how symptoms and side effects impact daily functioning) [5]. PROs are particularly relevant as an indicator of clinical benefit when new therapies have a modest impact on survival. PROs demonstrate significant associations with survival and other important outcomes, including performance status, adherence, and treatment discontinuation [8,9,10,11]. PRO data can also contribute to more accurate adverse event reporting in clinical trials, as data suggest that concordance between clinicians’ and patients’ reports of side effects is low [12,13]. PRO data collection gives a voice to patients, especially long-term survivors whose toxicity is generally not reported in clinical trials. Dissemination of PRO data can help future patients to make informed decisions about their care and contribute to shared decision-making [14].

Numerous phase III trials of ICIs have reported favorable quality of life in patients treated with ICIs relative to those treated with standard of care (e.g., chemotherapy). Nevertheless, there are notable omissions in the current literature. First, there are few published studies of PROs in patients treated with ICIs outside the context of a clinical trial [15,16,17,18,19]. This real-world evidence is important because patients treated on a clinical trial tend to be younger, healthier, and have higher socioeconomic status than those treated in the community setting [20,21]. In addition, clinical trials have reported almost exclusively on overall quality of life or disease-specific symptomatology (e.g., shortness of breath in lung cancer) but not patient-reported symptomatic toxicities of ICIs, such as diarrhea and rash. Omission of treatment-specific patient-reported toxicities may bias PRO findings against standard of care. The FDA noted the importance of studying treatment-specific PROs in its Guidance for Industry document in 2009 [22].

Patient registries administered by advocacy groups offer a way to rapidly address these knowledge gaps with little cost. Although population-based cancer registries are common, those developed by advocacy groups offer the opportunity to reach a subgroup of engaged patients to generate hypotheses to be tested in more representative clinical settings. In collaboration with the GO_2_ Foundation for Lung Cancer (previously the Bonnie J. Addario Lung Cancer Foundation), we prospectively collected PRO data from patients participating in the Lung Cancer Registry (www.lungcancerregistry.org) who reported receiving treatment with a PD-1 inhibitor (i.e., atezolizumab, durvalumab, nivolumab, pembrolizumab). The goals of the study were to: (1) evaluate the feasibility and acceptability of using patient registries to collect PRO data of novel anti-cancer agents, and (2) evaluate ICI-specific patient-reported toxicities and overall quality of life in patients treated with ICIs in the real-world setting. The study was intended to describe the overall patient experience rather than attribute PROs to a specific cause (e.g., treatment side effects versus disease symptomatology). Because the study was intended to be exploratory, no a priori hypotheses were generated.

## 2. Results

Data were collected between January 2018 and December 2019. In total, 258 of 646 (40%) of eligible patients responded to the survey invitation, of which 226 of 258 (87%) patients who started the survey completed it. Sociodemographic and clinical characteristics of the 226 participants are described in Table 1. The majority of participants were female (75%), White (90%), and from the United States (89%). Patients reported an average Charlson Comorbidity Index score of 3. Overall, 111 patients received pembrolizumab, 81 nivolumab, 29 durvalumab, and 13 atezolizumab, with eight patients reporting receipt of more than one. For a plurality of patients (47%), the duration of treatment with an ICI was 2–6 months. Due to toxicity, 25% reported a delay in treatment, 11% reported an emergency room (ER) visit, and 9% reported being admitted to the hospital. Patients treated with nivolumab reported significantly longer time on treatment than those treated with durvalumab or pembrolizumab and patients treated with atezolizumab reported significantly longer time on treatment than those treated with durvalumab (*p* < 0.001). Other sociodemographic and clinical characteristics did not significantly differ by ICI regimen (*p* values ≥ 0.07). Quality of life scores are also shown in Table 1. There were no group differences in overall quality of life or quality of life subscales at the time of assessment. Conversion of Functional Assessment of Cancer Therapy–General (FACT-G) subscale scores in the current sample to European Organisation for Research and Treatment of Cancer Quality of Life of Cancer Patients questionnaire (EORTC QLQ-C30) subscale scores yielded the following values: physical wellbeing (M = 73.86, 95%CI: 71.49 to 76.22), role/functional wellbeing (M = 56.61, 95%CI: 53.25 to 59.98), and emotional wellbeing (M = 57.23, 95% CI: 54.51 to 59.96) subscales. These values were significantly lower than those reported in the PACIFIC trial at 48 weeks post-initiation of durvalumab: physical functioning (M = 82.9), role functioning (M = 78.9), and emotional functioning (M = 84.8) [23].

Table 2 lists the frequency of symptomatic toxicities during treatment with ICIs. Those of any severity reported by a majority of the sample included fatigue (85%), aching joints (63%), aching muscles (57%), insomnia (56%), shortness of breath (51%), itching (50%), and skin dryness (50%). The three most common moderate-to-severe symptomatic toxicities were fatigue (41%), aching joints (27%), and aching muscles (20%).

## 3. Discussion

This registry-based study with the GO_2_ Foundation for Lung Cancer Foundation is among the first to our knowledge to report on PROs in lung cancer patients receiving ICIs outside the context of a clinical trial. Registry-based studies are an ideal way to engage patients in hypothesis-generating, quantitative studies. Hypotheses can then be tested in more representative patient samples. The current study demonstrated the feasibility and acceptability of a registry-based approach, with 226 patients recruited in 24 months and 87% of patients who started the survey completing it. Results indicated no differences by ICI regimen in overall quality of life or subscales. Study participants reported worse overall quality of life, emotional wellbeing, and functional wellbeing than U.S. population norms [24], as might be expected in advanced lung cancer patients. There were no differences in the physical and functional wellbeing of the sample compared to U.S. population norms. Interestingly, the sample also reported worse quality of life overall and in all domains compared to a normative sample of cancer patients [24]. These findings are consistent with studies in the pre-checkpoint era reporting greater supportive care needs and higher distress in advanced lung cancer patients compared to patients with other cancer diagnoses [25,26]. Quality of life in this study was also significantly worse than that reported at 48 weeks in a recent clinical trial of patients with lung cancer treated with durvalumab [23]. Our findings may be due to the sample composition; for example, perhaps registry participants have higher symptomatology than patients as a whole, particularly clinical trial participants. The current study also noted higher rates of any and moderate-to-severe symptomatic toxicities as compared to clinical trials. These findings are consistent with previous studies suggesting that provider-rated adverse events on clinical trials tend to underestimate PROs [27]. Although it has been widely noted that fatigue and rash are common symptomatic toxicities of ICIs [28,29], there has been less attention focused on arthralgias and myalgias. Data from studies of other patients with arthralgias and myalgias (e.g., in breast cancer patients treated with aromatase inhibitors) suggest that these side effects can be distressing and compromise daily functioning [30]. Thus, these toxicities deserve increased attention in the context of ICIs.

The current study is characterized by several strengths, including an international sample of real-world lung cancer patients that was heterogeneous in terms of ICI received, a validated measure of quality of life, and appropriate statistical analyses. Study limitations should also be noted, however. As this registry was not population-based, registry participants on the current study should not be considered representative of the larger population of advanced lung cancer patients treated with an ICI. Bias and non-representativeness may be exacerbated by putative non-representativeness of the subset of registry participants who completed the survey. Thus, it is unknown whether patients who did not respond to the survey invitation would have reported similar quality of life and immune-related adverse events to those patients who did provide data. Results are intended to be hypothesis-generating and should be investigated further in more representative patient samples. In addition, the cross-sectional survey asked about symptomatic toxicities during treatment with ICIs, which could have been current or completed months or years previously. Consistent with other studies of patient-reported outcomes, study participants may have been subject to recall bias that made it difficult to accurately remember what they were experiencing during treatment with ICIs. In this particular context, we believe retrospective data are valuable because they provide a first look at the prevalence of memorable (and likely impactful) symptomatic toxicities over the course of treatment. Thus, they provide important preliminary data to be further investigated in prospective, longitudinal studies. A related limitation is that patients were heterogeneous in terms of which current treatment they might be receiving. Thus, we were unable to partition the relative influence of long-term and late effects of ICIs on quality of life from the impact of current treatments. Findings of worse quality of life compared to U.S. population and cancer normative data suggest the importance of future follow-up studies to understand lung cancer survivorship after ICI treatment. In addition, because all data were reported by patients, we did not have access to electronic medical records that would provide additional clinical insight into patients’ experiences, e.g., tumor grade, number of metastatic sites, tumor progression, etc. Lastly, the sample was largely composed of White Americans. Additional studies are needed in diverse samples.

## 4. Materials and Methods

### 4.1. Participants

To be eligible, participants were: (1) age 18 or older, (2) diagnosed with lung cancer or a caregiver to a person diagnosed with lung cancer, (3) treated with or a caregiver to a patient treated with an immune checkpoint inhibitor (i.e., PD-1 or PD-L1), (4) enrolled or eligible to be enrolled in the Lung Cancer Registry, and (5) able to speak and read English. Because nearly all participants self-identified as patients, caregivers were not included in analyses.

### 4.2. Procedure and Measures

The study received a waiver of IRB approval from Advarra IRB, as no identifiable information was collected. Individuals self-reporting a diagnosis of lung cancer and treatment with a PD-1 or PD-L1 inhibitor who participated in the Lung Cancer Registry were invited to complete an online survey at one time point. Demographic information was obtained from participants through use of a standardized self-report questionnaire. Variables assessed included age, race, ethnicity, marital status, and education (see Appendix A). Severity of symptomatic toxicities during treatment with ICIs (e.g., fatigue, muscle pain, rash) was assessed using a 5-point Likert scale with 0 = not at all and 4 = very much [31]. Symptomatic toxicities were identified based on a review of the literature as well as qualitative interviews with 14 patients, 7 caregivers, and 9 providers. Quality of life at the time of assessment was evaluated with the FACT-G, a 27-item measure assessing physical wellbeing (PWB), functional wellbeing (FWB), social wellbeing (SWB), and emotional wellbeing (EWB) [32]. Items are summed to calculate a total score. Higher scores indicate better quality of life. Additional self-reported disease and treatment variables evaluated included comorbidities as assessed with the Charlson Comorbidity Index [33] (see Appendix A), months on ICI, ICI regimen, treatment delays due to toxicity, ER visits due to toxicity, and hospitalizations due to toxicity.

### 4.3. Statistical Analyses

Feasibility was defined as average patient recruitment per month. Acceptability was defined as percentage of patients starting the survey who completed it. Descriptive statistics were calculated using means, standard deviations, and percentages for sociodemographic and clinical characteristics. Group differences were examined using ANOVA or chi square tests, as appropriate. Participants’ FACT-G scores on the physical, functional, and emotional wellbeing subscales were transformed to corresponding EORTC QLQ-C30 subscale scores using published methodology [34] to compare quality of life in the current study to available data from clinical trials of ICIs in NSCLC [23]. Post hoc comparisons identified where any significant differences existed. Due to small cell sizes and to reduce the possibility of Type I error, groups were not compared on symptomatic toxicities. All data analyses were completed using SAS 9.4 (SAS Institute, Cary, NC, USA).

## 5. Conclusions

In summary, the results of the current study suggest that online patient registries should be considered to explore PROs of novel anti-cancer agents. More research is needed to validate patient-reported measures of symptomatic toxicities in this population and understand their prevalence and longitudinal course in prospective studies. Future prospective studies should also compare outcomes between patients on clinical trials and those treated in the community. In addition, providers should consider asking about common symptomatic toxicities that may be amenable to supportive care interventions. For example, physical activity has been shown to be beneficial in improving fatigue, arthralgias, and myalgias in other cancer populations, as well as contributing to better sleep [35,36]. Randomized trials of physical activity and other interventions to improve symptomatic toxicities of ICIs are also warranted.

## Figures and Tables

**Table 1:** Sociodemographic and clinical characteristics and patient-reported outcomes by immune checkpoint inhibitor received. Note: Some percentages do not equal 100 due to sporadic missing data.

Variable	All (*n* = 226)	Durvalumab (*n* = 29)	Nivolumab (*n* = 81)	Pembrolizumab (*n* = 111)	Atezolizumab (*n* = 13)	*p* Value
Age: M (SD) years	61.16 (10.52)	61.48 (9.13)	61.24 (10.29)	61.02 (10.79)	56.69 (10.00)	0.37
Gender: *n* (%) female	170 (75%)	23 (79%)	61 (75%)	81 (73%)	11 (85%)	0.76
Race: *n* (%) white	200 (90%)	27 (93%)	75 (94%)	94 (86%)	12 (92%)	0.37
Comorbidities: M (SD)	2.86 (1.62)	2.79 (0.94)	3.00 (1.75)	2.78 (1.65)	2.85 (1.52)	0.82
Time on Treatment: *n* (%)						<0.001
≤1 month	23 (10%)	3 (10%)	5 (6%)	14 (13%)	1 (8%)	
2–6 months	105 (47%)	18 (62%)	30 (37%)	54 (50%)	7 (58%)	
7–12 months	35 (16%)	7 (24%)	9 (11%)	21 (19%)	0 (0%)	
13–24 months	30 (14%)	1 (3%)	12 (15%)	17 (16%)	2 (17%)	
>24 months	29 (13%)	0 (0%)	25 (31%)	2 (2%)	2 (17%)	
Treatment delay due to toxicity: *n* (%)	56 (25%)	10 (34%)	20 (25%)	24 (22%)	3 (23%)	0.33
ER visit due to toxicity: *n* (%)	26 (12%)	2 (7%)	8 (10%)	15 (14%)	1 (8%)	0.82
Hospitalization due to toxicity: *n* (%)	20 (9%)	1 (3%)	4 (5%)	13 (12%)	2 (15%)	0.31
FACT-G Total: M (SD)	74.08 (17.28)	74.21 (19.96)	75.39 (16.21)	73.72 (16.86)	65.77 (16.30)	0.31
Physical	21.37 (5.68)	21.41 (5.13)	21.94 (5.73)	20.87 (6.04)	20.31 (6.51)	0.59
Functional	16.74 (6.34)	17.24 (7.05)	17.14 (5.63)	16.52 (6.57)	13.92 (6.01)	0.36
Emotional	16.39 (4.96)	16.72 (4.78)	17.35 (4.46)	15.98 (5.11)	13.92 (5.33)	0.07
Social	19.54 (6.02)	18.82 (8.00)	19.24 (5.73)	20.08 (5.44)	17.62 (6.96)	0.42
PROMIS Depression 4a: M (SD)	7.11 (3.14)	7.00 (3.23)	6.84 (2.91)	7.13 (3.28)	8.50 (2.94)	0.39

Note: Eight patients reported receipt of multiple immune checkpoint inhibitors. FACT-G: Functional Assessment of Cancer Therapy–General. Comorbidities represent the Charlson Comorbidity Index score. *p*-values correspond to ANOVA or chi-square tests of all groups, as appropriate.

**Table 2:** Most common symptomatic immune-related adverse events (irAEs). Note: Some percentages do not equal 100 due to sporadic missing data. Any irAE was defined as patients reporting “a little bit,” “somewhat,” “quite a bit,” or “very much.” A moderate to severe irAE was defined as patients reporting “quite a bit” or “very much.”

Symptom	Any Severity *n* (%)	Moderate-Severe *n* (%)	Missing *n*	Responses *n*
Fatigue	190 (85%)	92 (41%)	10	224
Aching joints	137 (63%)	58 (27%)	16	218
Aching muscles	122 (57%)	43 (20%)	21	213
Insomnia	122 (56%)	38 (18%)	17	217
Shortness of breath	109 (51%)	27 (13%)	20	214
Itching	108 (50%)	40 (18%)	16	218
Skin dryness	108 (50%)	38 (18%)	20	214
Back pain	103 (48%)	28 (13%)	19	215
Problems with memory	100 (47%)	18 (8%)	21	213
Problems with concentration	98 (46%)	14 (7%)	20	214
Numbness or tingling in hands or feet	98 (45%)	27 (12%)	17	217
Constipation	96 (44%)	26 (12%)	17	217
Cough (new or worsening)	94 (44%)	23 (11%)	19	215
Change in the way food tastes	86 (40%)	29 (13%)	19	215
Headache	85 (39%)	20 (9%)	18	216
Bone pain	84 (39%)	29 (14%)	21	213
Feeling bloated	83 (39%)	29 (14%)	20	214
Diarrhea	81 (38%)	25 (12%)	19	215
Frequent urination	80 (38%)	28 (13%)	22	212
Weakness in arms or legs	78 (37%)	18 (9%)	23	211
Nausea	79 (36%)	24 (11%)	17	217
Loss of appetite	77 (36%)	23 (11%)	21	213
Reflux or heartburn	77 (36%)	25 (12%)	22	212
Dizziness	74 (35%)	6 (3%)	22	212
Abdominal pain	73 (34%)	12 (6%)	22	212
Rash	72 (33%)	32 (15%)	18	216
Blurred vision	72 (34%)	14 (7%)	21	213
Problems with balance or coordination	70 (33%)	12 (6%)	19	215
Increased skin sensitivity to heat, cold, touch, or sunlight	67 (31%)	26 (12%)	19	215
Hives	65 (30%)	22 (10%)	19	215
Wheezing	63 (30%)	18 (8%)	21	213
Chest pain	61 (29%)	9 (4%)	21	213
Easy bruising or bleeding	59 (27%)	14 (6%)	18	216
Mouth sores	44 (20%)	9 (4%)	18	216
Shivering or shaking chills	41 (20%)	9 (4%)	24	210
Arm or leg swelling	36 (17%)	10 (5%)	24	210
Swelling in the face	27 (13%)	3 (1%)	22	212
Vomiting	18 (9%)	7 (3%)	27	207
Blood in stool	15 (7%)	3 (1%)	23	211
Vitiligo	10 (5%)	2 (1%)	23	211

## Data Availability

The data presented in this study are available on request from the corresponding author.

## Appendix A

Demographic and Comorbidity Questions Answered by Participants

Gender

Age

Ethnicity

State (USA Only)

Postal/Zip Code

Country

Race

- American Indian or Alaska Native
- Asian
- Black or African American
- Native Hawaiian or Other Pacific Islander
- White
- Unknown
- Other

Who is completing this survey?

What is the patient’s marital status?

What is the highest level of school the patient has COMPLETED?

What is the patient’s ethnic group?

Has the patient ever had a heart attack?

Has the patient ever been treated for heart failure?

Has the patient had an operation to unclog or bypass the arteries in his/her legs?

Has the patient had a stroke, cerebrovascular accident, blood clot or bleeding in the brain, or transient ischemic attack (TIA)?

IF YES, does the patient have difficulty moving an arm or leg as a result of the stroke or cerebrovascular accident?

Does the patient have asthma?

IF YES, does the patient take medicines for his/her asthma?

Does the patient have emphysema, chronic bronchitis, or chronic obstructive lung disease?

IF YES, does the patient take medicines for his/her lung disease?

Does the patient have stomach ulcers, or peptic ulcer disease?

IF YES, has this condition been diagnosed by endoscopy (where a doctor looks into the patient’s stomach through a scope) or an upper GI or barium swallow study (where the patient swallows chalky dye and then X-rays are taken)?

Does the patient have diabetes (high blood sugar)?

IF YES, has the diabetes caused problems with the patient’s kidneys?

IF YES, has the diabetes caused problems with the patient’s eyes, treated by an ophthalmologist?

Has the patient ever had the following problems with their kidneys?

- Poor kidney function (blood tests show high creatinine)
- Have used hemodialysis or peritoneal dialysis
- Have received kidney transplantation

Does the patient have rheumatoid arthritis?

IF YES, does the patient take medications for it regularly?

Does the patient have lupus (systemic lupus erythematosus)?

Does the patient have polymyalgia rheumatica?

Does the patient have any of the following conditions? (Select all that apply.)

- Alzheimer’s Disease, or another form of dementia
- Cirrhosis, or serious liver damage
- Leukemia or polycythemia vera
- Lymphoma
- AIDS
- Unsure

## References

[1] Gettinger S., Horn L., Jackman D., Spigel D., Antonia S., Hellmann M., Powderly J., Heist R., Sequist L.V., Smith D.C. (2018). Five-year follow-up of nivolumab in previously treated advanced non-small-cell lung cancer: Results from the CA209-003 study. J. Clin. Oncol..

[2] Garon E.B., Hellmann M.D., Rizvi N.A., Carcereny E., Leighl N.B., Ahn M.J., Eder J.P., Balmanoukian A.S., Aggarwal C., Horn L. (2019). Five-year overall survival for patients with advanced nonsmall-cell lung cancer treated with pembrolizumab: Results from the phase I KEYNOTE-001 study. J. Clin. Oncol..

[3] Garon E.B., Rizvi N.A., Hui R., Leighl N., Balmanoukian A.S., Eder J.P., Patnaik A., Aggarwal C., Gubens M., Horn L. (2015). Pembrolizumab for the treatment of non-small-cell lung cancer. N. Engl. J. Med..

[4] Herbst R.S., Baas P., Kim D.W., Felip E., Pérez-Gracia J.L., Han J.Y., Molina J., Kim J.H., Arvis C.D., Ahn M.J. (2016). Pembrolizumab versus docetaxel for previously treated, PD-L1-positive, advanced non-small-cell lung cancer (KEYNOTE-010): A randomised controlled trial. Lancet.

[5] Kluetz P.G., Slagle A., Papadopoulos E.J., Johnson L.L., Donoghue M., Kwitkowski V.E., Chen W.H., Sridhara R., Farrell A.T., Keegan P. (2016). Focusing on core patient-reported outcomes in cancer clinical trials: Symptomatic adverse events, physical function, and disease-related symptoms. Clin. Cancer Res..

[6] Di Maio M., Basch E., Bryce J., Perrone F. (2016). Patient-reported outcomes in the evaluation of toxicity of anticancer treatments. Nat. Rev. Clin. Oncol..

[7] Basch E., Abernethy A.P., Mullins C.D., Reeve B.B., Smith M.L., Coons S.J., Sloan J., Wenzel K., Chauhan C., Eppard W. (2012). Recommendations for incorporating patient-reported outcomes into clinical comparative effectiveness research in adult oncology. J. Clin. Oncol..

[8] Quinten C., Maringwa J., Gotay C.C., Martinelli F., Coens C., Reeve B.B., Flechtner H., Greimel E., King M., Osoba D. (2011). Patient self-reports of symptoms and clinician ratings as predictors of overall cancer survival. J. Natl. Cancer Inst..

[9] Eliasson L., Clifford S., Barber N., Marin D. (2011). Exploring chronic myeloid leukemia patients’ reasons for not adhering to the oral anticancer drug imatinib as prescribed. Leuk. Res..

[10] Atkinson T.M., Andreotti C.F., Roberts K.E., Saracino R.M., Hernandez M., Basch E. (2015). The level of association between functional performance status measures and patient-reported outcomes in cancer patients: A systematic review. Support. Care Cancer.

[11] Kadakia K.C., Snyder C.F., Kidwell K.M., Seewald N.J., Flockhart D.A., Skaar T.C., Desta Z., Rae J.M., Otte J.L., Carpenter J.S. (2016). Patient-reported outcomes and early discontinuation in aromatase inhibitor-treated postmenopausal women with early stage breast cancer. Oncologist.

[12] Atkinson T.M., Li Y., Coffey C.W., Sit L., Shaw M., Lavene D., Bennett A.V., Fruscione M., Rogak L., Hay J. (2012). Reliability of adverse symptom event reporting by clinicians. Qual. Life Res..

[13] Falchook A.D., Green R., Knowles M.E., Amdur R.J., Mendenhall W., Hayes D.N., Grilley-Olson J.E., Weiss J., Reeve B.B., Mitchell S.A. (2016). Comparison of patient—And practitioner-reported toxic effects associated with chemoradiotherapy for head and neck cancer. JAMA Otolaryngol. Head Neck Surg..

[14] Basch E. (2010). The missing voice of patients in drug-safety reporting. N. Engl. J. Med..

[15] Iivanainen S., Alanko T., Peltola K., Konkola T., Ekström J., Virtanen H., Koivunen J.P. (2019). ePROs in the follow-up of cancer patients treated with immune checkpoint inhibitors: A retrospective study. J. Cancer Res. Clin. Oncol..

[16] Dai W.F., Beca J., Croxford R., Isaranuwatchai W., Menjak I.B., Petrella T.M., Mittmann N., Earle C.C., Gavura S., Mercer R.E. (2020). Real-world, population-based cohort study of toxicity and resource utilization of second-line ipilimumab for metastatic melanoma in Ontario, Canada. Int. J. Cancer.

[17] Asher N., Ben-Betzalel G., Lev-Ari S., Shapira-Frommer R., Steinberg-Silman Y., Gochman N., Schachter J., Meirson T., Markel G. (2020). Real world outcomes of ipilimumab and nivolumab in patients with metastatic melanoma. Cancers (Basel).

[18] Joseph R.W., Liu F.X., Shillington A.C., Macahilig C.P., Diede S.J., Dave V., Harshaw Q., Saretsky T.L., Pickard A.S. (2020). Health-related quality of life (QoL) in patients with advanced melanoma receiving immunotherapies in real-world clinical practice settings. Qual. Life Res..

[19] Kandel M., Dalle S., Bardet A., Allayous C., Mortier L., Dutriaux C., Guillot B., Leccia M.T., Dalac S., Legoupil D. (2020). Quality-of-life assessment in French patients with metastatic melanoma in real life. Cancer.

[20] Unger J.M., Barlow W.E., Martin D.P., Ramsey S.D., Leblanc M., Etzioni R., Hershman D.L. (2014). Comparison of survival outcomes among cancer patients treated in and out of clinical trials. J. Natl. Cancer Inst..

[21] Gross C.P., Filardo G., Mayne S.T., Krumholz H.M. (2005). The impact of socioeconomic status and race on trial participation for older women with breast cancer. Cancer.

[22] U.S. Department of Health and Human Services (2018). Guidance for Industry Patient Reported Outcomes Measures: Use in Medical Product Development to Support. Labeling Claims. 19 October 2009. https://www.fda.gov/media/77832/download.

[23] Hui R., Özgüroğlu M., Villegas A., Daniel D., Vicente D., Murakami S., Yokoi T., Chiappori A., Lee K.H., de Wit M. (2019). Patient-reported outcomes with durvalumab after chemoradiotherapy in stage III, unresectable non-small-cell lung cancer (PACIFIC): A randomised, controlled, phase 3 study. Lancet Oncol..

[24] Brucker P.S., Yost K., Cashy J., Webster K., Cella D. (2005). General population and cancer patient norms for the functional assessment of cancer therapy-general (FACT-G). Eval. Health Prof..

[25] Sanders S.L., Bantum E.O., Owen J.E., Thornton A.A., Stanton A.L. (2010). Supportive care needs in patients with lung cancer. Psychooncology.

[26] Zabora J., BrintzenhofeSzoc K., Curbow B., Hooker C., Piantadosi S. (2001). The prevalence of psychological distress by cancer site. Psychooncology.

[27] Di Maio M., Gallo C., Leighl N.B., Piccirillo M.C., Daniele G., Nuzzo F., Gridelli C., Gebbia V., Ciardiello F., De Placido S. (2015). Symptomatic toxicities experienced during anticancer treatment: Agreement between patient and physician reporting in three randomized trials. J. Clin. Oncol..

[28] Wang Y., Zhou S., Yang F., Qi X., Wang X., Guan X., Shen C., Duma N., Vera Aguilera J., Chintakuntlawar A. (2019). Treatment-related adverse events of PD-1 and PD-L1 inhibitors in clinical trials: A systematic review and meta-analysis. JAMA Oncol..

[29] Michot J.M., Bigenwald C., Champiat S., Collins M., Carbonnel F., Postel-Vinay S., Berdelou A., Varga A., Bahleda R., Hollebecque A. (2016). Immune-related adverse events with immune checkpoint blockade: A comprehensive review. Eur. J. Cancer.

[30] Niravath P. (2013). Aromatase inhibitor-induced arthralgia: A review. Ann. Oncol..

[31] Webster K.A., O’Connor M.L., Hansen A.R., Kircher S., Jim H.S.L., Dicker A.P., Janda M., Ala-leppilampi K., Bingham C.O., Feliciano J. (2020). Development of a functional assessment of chronic illness therapy item library and primary symptom list for the assessment of patient-reported adverse events associated with immune checkpoint modulators. J. Cancer Metastasis Treat..

[32] Cella D.F., Tulsky D.S., Gray G., Sarafian B., Linn E., Bonomi A., Silberman M., Yellen S.B., Winicour P., Brannon J. (1993). The functional assessment of cancer therapy scale: Development and validation of the general measure. J. Clin. Oncol. Off. J. Am. Soc. Clin. Oncol..

[33] Charlson M.E., Pompei P., Ales K.L., MacKenzie C.R. (1987). A new method of classifying prognostic comorbidity in longitudinal studies: Development and validation. J. Clin. Dis..

[34] Holzner B., Bode R.K., Hahn E.A., Cella D., Kopp M., Sperner-Unterweger B., Kemmler G. (2006). Equating EORTC QLQ-C30 and FACT-G scores and its use in oncological research. Eur. J. Cancer.

[35] Chen Y.J., Li X.X., Ma H.K., Zhang X., Wang B.W., Guo T.T., Xiao Y., Bing Z.T., Ge L., Yang K.H. (2020). Exercise training for improving patient-reported outcomes in patients with advanced-stage cancer: A systematic review and meta-analysis. J. Pain Symptom Manag..

[36] Irwin M.L., Cartmel B., Gross C.P., Ercolano E., Li F., Yao X., Fiellin M., Capozza S., Rothbard M., Zhou Y. (2015). Randomized exercise trial of aromatase inhibitor-induced arthralgia in breast cancer survivors. J. Clin. Oncol..

